# Using Organoids to Model Sex Differences in the Human Brain

**DOI:** 10.1016/j.bpsgos.2024.100343

**Published:** 2024-06-04

**Authors:** Adam Pavlinek, Dwaipayan Adhya, Alex Tsompanidis, Varun Warrier, APEX Consortium, Simon Baron-Cohen, Carrie Allison, Varun Warrier, Alex Tsompanidis, Dwaipayan Adhya, Rosie Holt, Paula Smith, Tracey Parsons, Joanna Davis, Matthew Hassall, Daniel H. Geschwind, Alexander EP. Heazell, Jonathan Mill, Alice Franklin, Rosie Bamford, Jonathan Davies, Matthew E. Hurles, Hilary C. Martin, Mahmoud Mousa, David H. Rowitch, Kathy K. Niakan, Graham J. Burton, Fateneh Ghafari, Deepak P. Srivastava, Lucia Dutan-Polit, Adam Pavlinek, Madeline A. Lancaster, Ilaria Chiaradia, Tal Biron-Shental, Lidia V. Gabis, Anthony C. Vernon, Madeline Lancaster, Jonathan Mill, Deepak P. Srivastava, Simon Baron-Cohen

**Affiliations:** h Autism Research Centre, University of Cambridge, Cambridge, United Kingdom; i UCLA, Los Angeles, California; j Tommy’s Maternal and Fetal Research Centre, University of Manchester, Manchester, United Kingdom; k University of Exeter Medical School, College of Medicine & Health, University of Exeter, Exeter, United Kingdom; l Wellcome Trust Sanger Institute, Hinxton, United Kingdom; m Department of Pediatrics, University of Cambridge, Cambridge, United Kingdom; n Centre for Trophoblast Research, University of Cambridge, Cambridge, United Kingdom; o MRC Centre for Neurodevelopmental Disorders, King’s College London, London, United Kingdom; p MRC Laboratory of Molecular Biology, University of Cambridge, Cambridge, United Kingdom; q Department of Obstetrics and Gynecology, Meir Medical Center, Israel; r Child Development Division, Maccabi Health Services, Israel; a Department of Basic and Clinical Neuroscience, Institute of Psychiatry, Psychology and Neuroscience, King’s College London, London, United Kingdom; b MRC Centre for Neurodevelopmental Disorders, King’s College London, London, United Kingdom; c Autism Research Centre, Department of Psychiatry, University of Cambridge, Cambridge, United Kingdom; d Wellcome-MRC Cambridge Stem Cell Institute, University of Cambridge, Cambridge, United Kingdom; e Department of Psychiatry, University of Cambridge, Cambridge, United Kingdom; f MRC Laboratory of Molecular Biology, Cambridge, United Kingdom; g Department of Clinical and Biomedical Sciences, Faculty of Health and Life Sciences, University of Exeter, Exeter, United Kingdom

**Keywords:** Autism, Brain organoids, Sex chromosomes, Sex differences, Steroids, X chromosome inactivation

## Abstract

Sex differences are widespread during neurodevelopment and play a role in neuropsychiatric conditions such as autism, which is more prevalent in males than females. In humans, males have been shown to have larger brain volumes than females with development of the hippocampus and amygdala showing prominent sex differences. Mechanistically, sex steroids and sex chromosomes drive these differences in brain development, which seem to peak during prenatal and pubertal stages. Animal models have played a crucial role in understanding sex differences, but the study of human sex differences requires an experimental model that can recapitulate complex genetic traits. To fill this gap, human induced pluripotent stem cell–derived brain organoids are now being used to study how complex genetic traits influence prenatal brain development. For example, brain organoids from individuals with autism and individuals with X chromosome–linked Rett syndrome and fragile X syndrome have revealed prenatal differences in cell proliferation, a measure of brain volume differences, and excitatory-inhibitory imbalances. Brain organoids have also revealed increased neurogenesis of excitatory neurons due to androgens. However, despite growing interest in using brain organoids, several key challenges remain that affect its validity as a model system. In this review, we discuss how sex steroids and the sex chromosomes each contribute to sex differences in brain development. Then, we examine the role of X chromosome inactivation as a factor that drives sex differences. Finally, we discuss the combined challenges of modeling X chromosome inactivation and limitations of brain organoids that need to be taken into consideration when studying sex differences.

## Plain Language Summary

Sex differences are a contributing factor in neuropsychiatric conditions such as autism, which is more prevalent in males. Sex differences occur through interactions between sex steroid hormones such as estrogen and testosterone and sex chromosomes (chrX and chrY). Human stem cell–derived brain organoids are laboratory models that mimic brain development. For example, in individuals with neurodevelopmental conditions, brain organoids have revealed an imbalance of neuron populations compared with neurotypical individuals. In this review, we discuss sex steroid and sex chromosome influences on brain development and challenges of this model that need to be taken into account when studying sex differences.

Steroid hormones such as estrogens, androgens, and progestins that regulate sex-based development of tissues and organs (henceforth called sex steroid hormones) and the sex chromosomes (chromosomes X [chrX] and Y [chrY]) are known to influence sex differences in brain development, both independently and through their interaction (1, 2, 3). Together, they are responsible for global effects on behavior, neuroanatomy, and cellular and molecular mechanisms (4, 5, 6, 7). Independent human behavioral studies have identified sex differences in language, literacy, social skills, and empathy (8). Human sex differences are also well established in neuropsychiatric conditions such as autism (9), attention-deficit/hyperactivity disorder (9), and early-onset obsessive-compulsive disorder (10, 11, 12).

There is considerable evidence in humans of neuroanatomical differences between males and females. Brain volume, which is a global measure of structural differences, is larger in males than females on average (13, 14, 15). However, gray matter density is higher in females across the cerebral cortex (16,17). Sex differences have also been found in specific brain regions. Large studies have reported sex differences in hippocampal and amygdala volumes (18, 19, 20), and the relative size differences between males and females in both structures have been found to depend on age and pubertal development. Females showed larger hippocampal volumes postpuberty (19), while males showed overall larger amygdala volumes during both youth and adulthood (18). Although considerable variation has been reported in size differences between male and female cortical substructures, all studies have consistently reported the existence of sex differences in age-related developmental trajectories (18, 19, 20, 21). Sex differences have also been found in noncortical regions; in the cerebellum, females have larger cerebellar lobules such as Crus II connected with nonmotor regions of the cortex while males have larger motor region–connected lobules (H V and H VIIIA/B) (22).

However, understanding of molecular mechanisms that influence sex differences is predominantly informed by animal models (23, 24, 25). Because most sex differences in human behavioral phenotypes are more complex and often diverge from those of other mammals (26), there is a need for human-specific model systems to study mechanisms. Human-derived stem cells including embryonic stem cells and induced pluripotent stem cells (iPSCs) are able to capture unique human phenotypes driven by complex genetic polymorphisms. Because iPSCs are derived by the reprogramming of cells from individuals who carry specific polymorphisms associated with health and disease (27), in theory, this model enables direct correlation of individual behavioral traits with the molecular mechanisms conferred by their genetic background (28). By growing stem cell–derived, 3-dimensional (3D), self-organizing, and self-patterning neural organoids (hereafter referred to as brain organoids) (29), it is possible to study aspects of molecular mechanisms associated with human brain development and the factors that may disrupt them.

In this review, we focus on the biological basis of sex differences in the brain, specifically highlighting the cellular and molecular mechanisms involved and how human brain organoids are being used to dissect these mechanisms. First, we explore the nature of sex differences and how sex steroid hormones and sex chromosomes act as biological drivers. Then, we take an in-depth look into sex chromosomal regulatory mechanisms that are less well understood in the context of sex differences. Then, we discuss the advantages of using a human stem cell–derived brain organoid model to study sex differences in cellular and molecular traits. Finally, we discuss limitations of using stem cells and brain organoids in the context of developmental phenotypes associated with sex differences.

## Framework and Biological Drivers of Sex Differences in Human Behavior

Sex differences cannot be defined strictly based on their biological mechanisms. It is generally believed that sex steroid hormones and sex chromosomes are evolutionarily selected biological factors that influence sex differences (henceforth, we will call these factors biological drivers) (30). However, for reasons of experimental design, scientists have categorized sex differences into 4 broad types: qualitative, quantitative, latent, and population-level, with evidence of these 4 types found in both humans and animals (30,31) (Table 1). These sex differences may be a result of gonadal sex steroids that cause early-life programming during a sensitive prenatal period of brain development and during an additional sensitive period during puberty (32). These sex differences may also be the result of unequal activity of chrX and chrY driving sex differences independent of the gonads and long before their formation, and there is evidence of sexually differentiated gene expression by the 8-cell embryo stage (33).

**Table 1:** Framework for Sex Differences in Human Behavior

Sex Differences Classification	Description	Example
Qualitative	Sexual dimorphisms related to reproduction (143)	During spatial navigation performance tasks, men tended to rely more on geometric knowledge while women relied on rote learning (144).
Quantitative	Average sex differences, occurring on a continuum with a different mean for males and females or where male and female traits differ in the size of their response (145)	In acute and chronic pain response, men show greater sensitivity than women on average (145).
Latent	Hidden sex differences that are less well understood, but where the end point traits in males and females are the same, but there are underlying mechanistic differences that may only appear during environmental challenges such as stress, injury, or disease (146)	During eye-blink conditioning, male and female rats do not demonstrate sexual dimorphism in performance. However, performance in male rats is significantly improved upon response to stress, whereas the same stress response impairs performance in females (147).
Population-Level	Reflects differences in the distribution of individual traits (46)	In autism, representative behavioral traits are the same in males and females, but there is a skewed male-to-female prevalence of autistic traits (46).

However, our understanding of how these biological drivers influence molecular mechanisms is informed primarily by animal studies (34), and animal models do not always resemble humans because there is significant species-dependent divergence in brain development (35, 36, 37). For example, overall brain size and connectivity is greater in humans than in rodents and other primates. The human cerebral cortex and cerebellum are significantly larger than those of other primates (38). Proportions of neuronal populations are different in humans; 40% of neurons in humans are upper-layer pyramidal neurons compared with 25% in mice (39). Interneuron numbers are also higher in humans (25%–30%) than in rodents (∼20%) (40). Some human cortical circuits are driven by unique genetic modifiers called human accelerated regions that are not found in rodents (37). Human accelerated regions are regulatory elements on the genome that are enriched for genes that influence neural and cortical development (41). Even steroid hormone response varies between humans and rodents; for example, in humans, androgens are responsible for masculinization of the brain, while in rodents, this is influenced by estrogen (42). Brain size, connectivity, and population of neuronal types are all biological markers influenced by sex differences, as we will discuss in later sections.

## Biological Drivers of Sex Differences Are Also Autism Likelihood Factors

The biological drivers of sex differences—sex steroid hormones and sex chromosome complement—are associated with neuropsychiatric conditions. Autism is a typical example of a neuropsychiatric condition with behavioral sex differences (43). Current estimates suggest a 3:1 male-to-female ratio even after accounting for diagnostic bias (44, 45, 46). Atypical prenatal sex steroid levels are associated with increased prevalence of autism and autistic traits (47,48), with both testosterone and estrogen found to be elevated during the critical period of prenatal development in boys with autism (47,49). In females, a higher burden of genetic mutations has been shown to be associated with autism and autistic traits (50,51). This suggests that the combination of sex steroids and genes may increase the likelihood of autism in males. Gene dosage from sex chromosomes are believed to contribute to brain sex differences and autism likelihood (52) (Table 2). For example, the number of autistic traits is increased in individuals with sex chromosomal aneuploidies such as Klinefelter syndrome (KS) (XXY, 47), XYY syndrome (XYY, 47), and Turner syndrome (TS) (XO, 45) (53). Rett syndrome, where there is a mutation in the X-linked gene *MECP2* (54), and fragile X syndrome, with a mutation in the X-linked *FMR1* gene, are two of the most common syndromic forms of autism (55). Mutations in X-linked genes such as *KDM5C* (56), *ARHGEF9* (57), and *SYN1* (58) have recently been shown to confer a high likelihood of autism. According to the SFARI gene database, a total of 22 X-linked genes are classified as high-likelihood autism genes (https://gene.sfari.org/database/human-gene/).

**Table 2:** Genotype-Phenotype Relationships of X-Linked Conditions in Humans

X-Linked Condition	Genotype	Sex Bias	Cognitive Symptoms	Evidence From Brain Organoids
Rett Syndrome	Heterozygous mutations in *MECP2*	Males die before birth or early infancy	Autism, epilepsy (148)	Altered interneuron populations in MECP2 mutant, with more VIP^+^ and CALB2^+^ cells in the mutant Increase in density of excitatory puncta in the mutant Recurring epileptiform-appearing spikes and high-frequency oscillations (120)
FXS	Heterozygous mutations in *FMR1* gene	Females show milder symptoms	Intellectual disability, autism (149)	Accelerated neural progenitor cell cycle exit in FXS Reduced neural progenitor cell proliferation Number of GABAergic neurons decreased in FXS Accelerated synapse formation and hyperexcitability of synapses (121)
Klinefelter Syndrome	Males with extra X chromosome	Only affects males	Reading disabilities, autism (113,150)	Cellular phenotypes unknown. No evidence from brain organoids.
Turner Syndrome	Females missing part or all of 1 X chromosome	Only affects females	Intellectual disability, visual-spatial and cognitive weaknesses, autism (151,152)	Cellular phenotypes unknown. No evidence from brain organoids.

FXS, fragile X syndrome.

## Molecular Pathways Conferring Steroid- and Sex Chromosome–Mediated Sex Differences in the Brain

McCarthy and Arnold (25) have proposed 2 models of sexual differentiation, the classic model and the parallel model. According to the classic model, sex differences in brain and behavior are due to the developmental action of gonadal sex steroids. According to the parallel model, sex differences are the result of an imbalance of genes on the sex chromosomes, which suggests a more direct influence of X and Y chromosomal genes on sex differences.

**Figure 1:**
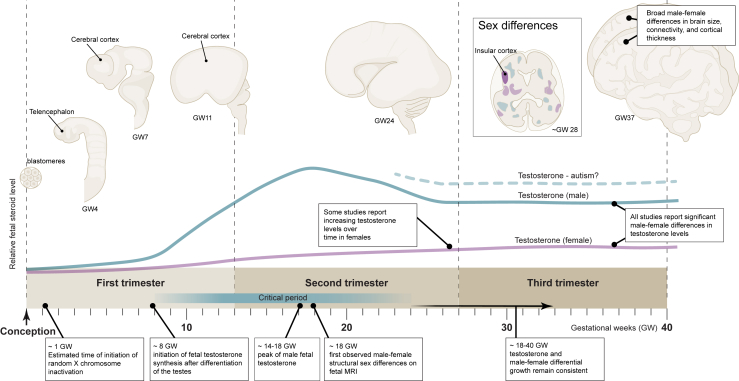
Timeline of human brain development from conception to birth showing human brain morphology over developmental time (top), predicted testosterone dynamics (middle), and key events in sex-biased development (bottom). The box shows an axial section with examples of regions with growth trajectory (blue, male-biased; purple, female-biased) sex differences found in fetal MRI (141). The insular cortex is an example of a focal region that was larger in males than in females. The sex chromosomes begin exerting sex-specific effects soon after conception. Testosterone is given here as a classic example of a steroid sex hormone with levels that differ by sex. Fetal testosterone increases after the differentiation of the testes in males. Male testosterone is thought to peak between GW14 and GW18 and then remain relatively consistent (141). Studies have generally reported higher male testosterone, and some studies have reported increasing testosterone in females over time (142). GW, gestational week; MRI, magnetic resonance imaging.

Although present in both males and females, steroid hormone levels usually differ by sex (Figure 1) (59,60). During development, sex steroids originate from the placenta and the maternal circulation (61), the fetal gonads (testes in males), and the brain (25). Most steroid hormone receptors are transcription factors that directly regulate gene activity; however, receptors for estrogens and androgens may also act via second messenger pathways. In the brain, estradiol is known to bind with ER⍺ and ERβ (estrogen receptors ⍺ and β), which act as transcription factors while second messenger pathways are mediated through GPER1 (G protein–coupled estrogen receptor 1) (62,63). Much less is known about the mechanism of androgen activity in the brain. Androgens are known to act solely through the X-linked androgen receptor, with well-established transcription factor activity and second messenger activity that is less well understood (64).

**Figure 2:**
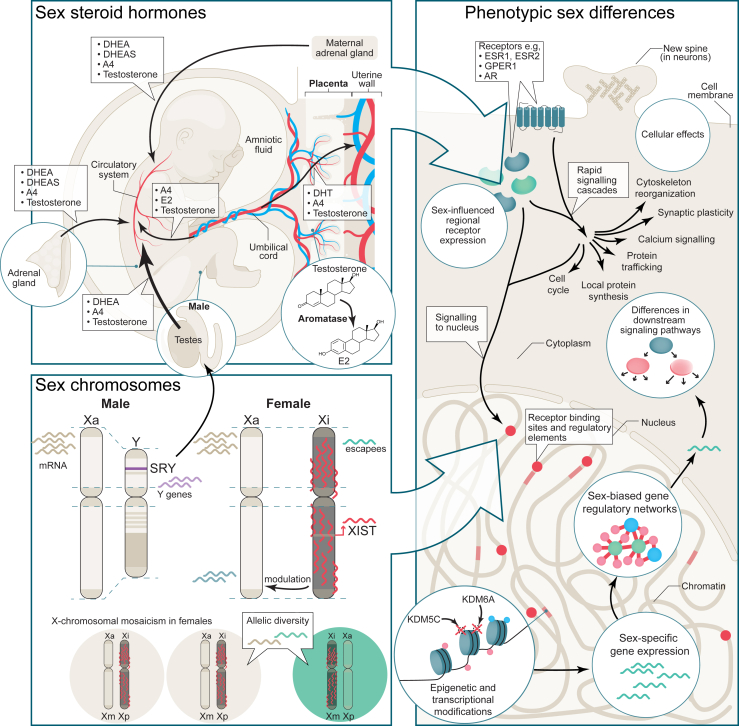
Sex steroid hormones from various sources (top left) and sex chromosome effects (bottom left) give rise to phenotypic sex differences at the cellular level. Note that although we show the placenta in the context of hormones, the placenta can influence sex differences both through sex steroid hormones or sex chromosome complement. Sex steroid hormones in the fetus can originate from the maternal adrenal gland, the placenta, and from the fetal adrenal gland. After entering the fetal circulatory system, hormones can cross the blood-brain barrier to affect brain development. In males, the testes develop and are a source of androgens. DHEA, DHEAS, A4, E2 are examples of key steroid sex hormones. Aromatase (bottom right) can aromatize testosterone to E2. The sex chromosomes (bottom left) can be a source of gene expression differences. In males, there is 1 active X chromosome and the Y chromosome. The *SRY* gene on the Y chromosome is responsible for testes development. Expression of other Y genes (purple) that have diverged from their X chromosome homologs in evolution can be a source of sex differences. Females have 1 Xa and 1 Xi. Sex-specific gene expression can originate from genes that are expressed from the Xi (escapees) or from modulation of gene expression on the Xa by the Xi. Finally, X-chromosomal mosaicism in females results in allelic diversity (bottom). The placenta is one example of a tissue where sex chromosome complement has an important role in sex differences. Examples of how various cellular and molecular brain sex differences may arise are shown (right). Exposure to steroid hormones can have rapid cellular effects mediated by rapid signaling cascades or canonical gene expression effects through nuclear signaling. Sex chromosome gene expression effects combined with gene expression in response to steroid hormone signaling can modulate regional receptor expression and steroid signaling pathways, resulting in further sex-specific cellular responses. The combined action of the sex chromosomes and steroid hormones can establish sex-biased gene expression, gene regulatory networks, and epigenetic modifications. One example of epigenetic modifications is demethylation, such as by the KDM5C and KDM6A demethylases, which are discussed. A4, androstenedione; AR, androgen receptor; DHEA, dehydroepiandrosterone; DHEAS, dehydroepiandrosterone sulfate; E2, estradiol; ESR, estrogen receptor; GPER1, G protein–coupled estrogen receptor 1; Xa, active X chromosome; Xi, inactive X chromosome; Xm, X maternal; Xp, X paternal.

There is now growing appreciation that the sex chromosome complement influences sex differences more directly (25,65) (Figure 2). In humans, most tissues show sex-biased gene expression in a subset of sex chromosomal and autosomal genes (66,67). One study found that there was more male-biased gene expression during prenatal brain development (67). The researchers observed changes in sex-differential gene expression based on developmental stage, where a subset of chrY genes (∼12 genes: *KDM5D*, *DDX3Y*, *ZFY*, *PCDH11Y*, *USP9Y*, *RPS4Y1*, *CYorf15B*, *TMSB4Y*, *NLGN4Y*, *UTY*, *EIF1AY*, and *GYG2P*) appeared to be consistently expressed over time while chrX genes seemed to follow a dynamic expression pattern (67). For example, via their histone demethylase activity, *KDM5C* and *KDM6A* were identified as major X-linked mediators of sex differences, and the *XIST* gene mediated X chromosome inactivation (XCI), thereby offsetting an imbalance in gene expression in females versus males (68,69). Associated with prenatal development, the X-linked *OGT* expressed in the placenta was found to have an indirect but significant effect on the brain (70) (Figure 2). Sex chromosomes may also exert influence through orthologs (genes that have evolved from a common ancestor during speciation) of chrX genes that have been retained on chrY during evolution. For example, *KDM5C* on chrX and *KDM5D* on chrY have subtle functional differences (71). The role of XCI, which regulates X-linked gene activity such as that of *KDM5C* and *KDM6A* described above (52), will be discussed in more detail in later sections. In the following section, we will summarize the cellular phenotypes affected as a result of sex steroid and sex chromosome activity.

## Effect of Sex Steroids and Chromosomal Sex on Cellular Phenotypes in the Brain

A number of studies that have investigated sex steroid effects have shown marked hormone-mediated sex differences in the brain, specifically in the hippocampus (72, 73, 74). Androgens (both testosterone and dihydrotestosterone) have been found to increase dendritic spine density (73,75,76) while also influencing synaptic long-term depression via a postsynaptic mechanism (77,78). Estrogens have also been shown to increase spine density but have been observed to influence synaptic long-term potentiation (72,79, 80, 81), and this was more prominent in females than males (79,80). In the hypothalamus, estrogen increased survivability of GABAergic (gamma-aminobutyric acidergic) neurons (82,83) and GABAergic projection size in males (84). A wide range of studies that have investigated sex steroid-mediated cellular and molecular mechanisms are summarized in Table S1.

There is accumulating evidence that chromosomal sex may also influence cellular traits independent of effects mediated through sex steroids (25). Mouse models that allow for the separation of gonadal sex and sex chromosomes by the deletion of the *Sry* gene from chrY and insertion of a *Sry* transgene into an autosome (known as the four core genotypes model) (2) have found that the presence of only 1 chrY or the absence of 2 chrXs irrespective of gonadal sex increased male bias in a maternal antibody–induced model of autism (85). More sex steroid–independent mechanisms were found using four core genotype mice; for example, XY hypothalamic neurons had greater axonal length than XX hypothalamic neurons (86), while higher numbers of dopaminergic neurons were observed only in male neuronal cultures (87). Beyond the four core genotypes model, hippocampal neurons have been found to develop larger pools of synaptic vesicles in male versus female rats via a sex steroid–independent mechanism (88). Nonneuronal cells such as astrocytes located in the arcuate nucleus of the hypothalamus showed more complex stellate features only in female rats (89). Microglia also showed sex differences in rodents such as in response to environmental factors such as microbiota (90).

## XCI Plays a Unique Role in Mediating Sex Chromosomal Effects

Sex chromosomes may have additional influences on sex differences via the X-linked gene regulatory mechanism known as XCI. XCI is a biological process that typically silences one of the 2 chrXs in females (XX individuals) (91). Males (XY individuals) only have 1 chrX, which is always active. Most genes on the inactivated chrX (Xi) are silent, except for ∼20% of Xi genes, which escape XCI on the pseudoautosomal regions of chrX, resulting in higher expression of these genes in XX individuals than in XY individuals (92). Xi modulates gene expression both in cis (i.e., genes found on the same Xi chromosome) and in trans genes (i.e., genes on the other chrX); this means that in XX individuals, Xi is likely to have an influence on gene expression from the active chrX (Xa) (52).

**Figure 3:**
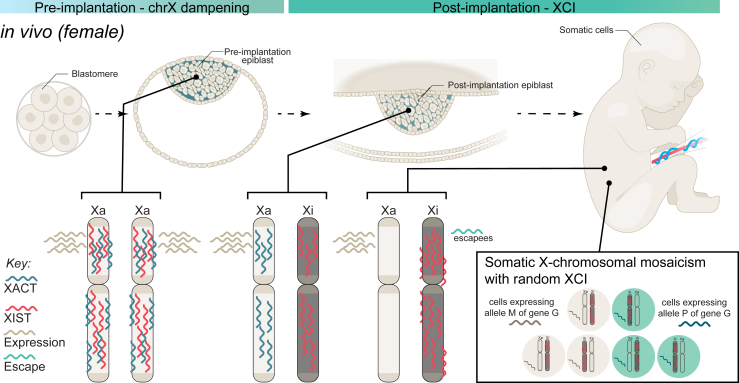
Typical chrX states in female human embryo development. Human genome activation and expression from the X chromosomes occurs by the 8-cell stage (far left). In the preimplantation embryo (left), gene expression occurs from both of the X chromosomes, which are coated with the long noncoding RNAs XIST and XACT. Gene expression is modulated through chrX dampening. Sometime after implantation (middle), random XCI occurs. Gene expression becomes restricted to only Xa, and Xa is coated with XACT. Xi is coated with XIST, and gene expression is silenced. In somatic cells, Xi remains silenced except for escapee genes. Random XCI is preserved in somatic cells (box), resulting in X-chromosomal mosaicism in females, where a subset of cells will express the maternal allele of a gene, and the remaining cells will express the paternal alleles of a gene. chrX, X chromosome; G, gene; M, maternal; P, paternal; Xa, active chrX; XCI, X chromosome inactivation; Xi, inactive chrX.

XCI is first seen following implantation of the embryo in humans, and inactivation of chrX occurs randomly so that each chrX is active in approximately half of all cells (93,94) (Figure 3). This phenomenon is known as X-chromosomal mosaicism, and XCI in these cells are stably maintained down cell lineages (95). The chrX-linked long noncoding RNA *XIST* is responsible for XCI initiating a dampening of transcription via the reduction of gene-activating histone marks and a concurrent increase in gene-repressive histone marks (96, 97, 98). This process is triggered by *XIST* RNA spreading over cis-regulatory regions across the X chromosome, where it recruits a wide range of chromatin-modifying factors (98,99). The Xa is also transiently coated by a long noncoding RNA, *XACT*, which interacts with *XIST* antagonistically to maintain chrX activity (100). It is essential for the survival of the embryo that *XIST* expression be monoallelic, and a region on the X chromosome called the X inactivation center maintains this monoallelic expression of *XIST* (90,101).

Non-pseudoautosomal genes of chrX provide a causal link between XCI and sex differences in the brain. For example, for non-pseudoautosomal chrX genes with chrY orthologs, the brain developmental genes *NLGN4X* and *ZFX* showed similar expression levels in males and females, but expression of the orthologous *NLGN4Y* and *ZFY* that only occurred in males offset an overall higher dosage of the NLGN4 and ZF gene species in males (102). Although studies have shown that alteration of a single residue in *NLGN4Y* hindered its ability to mediate synaptogenesis (103), the likelihood of autism, which was maintained through several generations, only appeared to increase significantly upon a mutation in *NLGN4X*. This resulted in greater susceptibility in males than females who carried a nonmutant *NLGN4X* allele (104).

## The Role of XCI in X-Chromosomal Aneuploidies

X-chromosomal aneuploidies in humans have revealed the significance of XCI in brain development. In the case of TS (45, XO), the most common female sex chromosome aneuploidy, 99% of pregnancies spontaneously terminate within the first trimester (105). In children with TS, it was found that the single chrX did not undergo inactivation, and there was downregulation of pseudoautosomal and XCI escape genes (106). Another study observed that TS-associated embryonic lethality was a result of haploinsufficiency of pseudoautosomal genes (107). The 1% who survived to term demonstrated significant mental and physical disabilities (105). These include deficits in visual-spatial functions and social skills and comorbidities such as attention-deficit/hyperactivity disorder and autism (105). In KS (47, XXY), the most common male sex chromosome aneuploidy, the presence of an additional X chromosome results in variable symptomatology, with only an estimated 25% of XXY genotypes being diagnosed with KS (108,109). This was believed to be due to the additional X chromosome being inactive and variable gene expression of pseudoautosomal escapee genes from this chromosome (108,110). Cognitive symptoms associated with KS were found to be driven by hypogonadism and included social impairments and verbal deficits and co-occurring conditions such as learning disability, autism, and epilepsy (108,111, 112, 113, 114, 115). A recent study identified a set of ∼12 genes dysregulated as a result of the additional Xi in individuals with KS; this included XCI escape genes (*KDM6A*, *KDM5C*, *SMC1A*, *ZFX*, *RBBP7*, *DDX3X*, *CDK16*, *DLG3*, *USP9X*, and *BCOR*) and pseudoautosomal genes (*SLC25A6* and *SHOX*) (52). However, the XCI and chrX gene dosage–mediated mechanisms associated with chrX aneuploidies remain mostly unknown.

## Brain Organoids Can Be Used to Study Biological Drivers of Sex Differences

**Figure 4:**
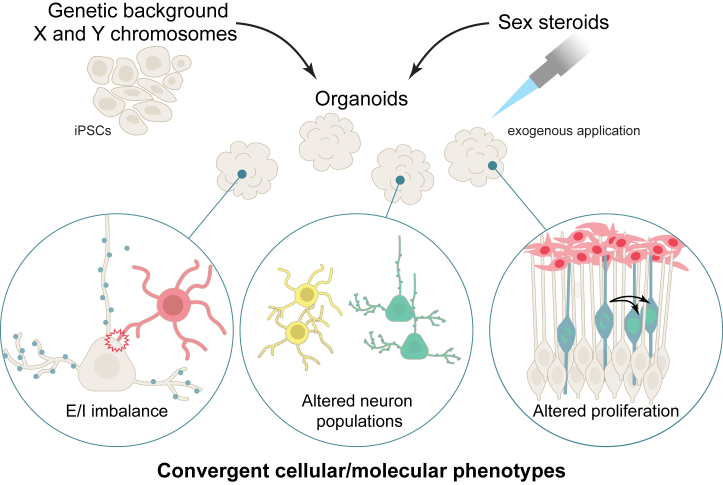
Examples of convergent cellular and molecular neuropsychiatric condition phenotypes revealed by studies using human iPSC-derived brain organoids: E/I imbalance, altered cell proliferation, and phenotypes that affect specific neuron classes. Future organoid studies can combine the study of genetic background, sex chromosome complement, and exogeneous steroid hormone application. E/I, excitation/inhibition; iPSC, induced pluripotent stem cell.

Brain organoids have been reliable models for studying cell proliferation and excitation-inhibition (E-I) imbalance and cellular phenotypes affected in neuropsychiatric conditions (35) (Figure 4). Mariani *et al.* showed increased proliferation of GABAergic neurons in iPSC-derived brain organoids from individuals with autism giving rise to atypical neuronal hyperpolarization associated with Na_v_1.1, a sodium channel preferentially expressed in GABAergic neurons (116). In another study that examined mutations in 3 high-likelihood autism genes (*SUV420H1*, *ARID1B*, and *CHD8*), the authors found that mutations in any one of these genes resulted in atypical development of GABAergic interneurons and excitatory projection neurons but through distinct molecular pathways (117), and this resulted in a reduction in the frequency and duration of spontaneous activity of neurons resembling network activity associated with the developing brain. More advanced brain organoid systems such as air-liquid interface cortical organoids (118) and cortical spheroids (119) capable of achieving greater neuronal maturity can effectively model E-I phenotypes associated with later prenatal and early postnatal periods.

Mutations in X-linked genes such as *MECP2* (which causes Rett syndrome) and *FMRP*, which causes fragile X syndrome, present with more severe neuropsychiatric symptoms in males than in females, and Rett syndrome is usually lethal in males, with only a few males known to have survived until infancy (54,55). In Rett syndrome brain organoids, the electrophysiological activity of inhibitory interneurons was significantly reduced compared with controls, even though no differences were found in the ratio of glutamatergic to GABAergic neuronal fates (120). In this case, E-I imbalances were attributable to altered neuronal fate. In fragile X syndrome, loss of the *FMRP* gene triggered a reduction in global proliferation rate in brain organoids coupled with premature neural differentiation (121). This produced fewer inhibitory neurons, which resulted in increased excitability of excitatory neurons. In typical brain organoids from males and females without mutations in X-linked genes, administration of elevated levels of sex steroids was found to increase glutamatergic (excitatory) neuronal output via increased cell proliferation, suggesting another mechanism for E-I imbalances (122). The ability of brain organoids to model neuronal activity associated with neuropsychiatric conditions and demonstrate atypical cellular phenotypes in X-linked conditions and in elevated steroid level conditions adds to their validity as a model for studying brain sex differences in humans.

## Limitations of Stem Cell Models Due to Atypical XCI States

Sex chromosomes and XCI play a key role in typical brain development (108). XCI is first observed during implantation of the embryo (93). In theory, stem cell models such as human embryonic stem cells or iPSCs may be most suited to studying the effect of XCI during human brain development. However, few studies have explored XCI traits using stem cell models. This is because in vitro embryonic stem cells and iPSCs demonstrate atypical XCI states that do not resemble in vivo XCI states (123). Four in vitro XCI states are known to exist: 1) Xi^XIST+^Xa, where 1 X chromosome is coated by XIST and is inactive; 2) XiXa, where the inactive X is not coated with XIST; 3) XaXa, where both X chromosomes are active; and 4) XeXa, where the Xi has undergone erosion (Xe) or a partial loss of inactivation (123).

While in humans Xi^XIST+^Xa is the typical XCI state, the XiXa state is functionally similar to Xi^XIST+^Xa, although it is not observed in vivo (124). XaXa and XeXa represent atypical XCI states of full or partial reactivation, respectively, of the inactive chrX due to Xi erosion, and they cannot be reversed during differentiation (125,126), which results in a permanent burden of atypical chrX gene expression during the lifetime of differentiated cells. XCI mosaicism, which balances allelic expression in case of heterozygous disease-causing X-linked nonsynonymous mutations, also cannot be achieved in these stem cell models because they maintain clonal X activation status (125). The Xi erosion–induced atypical XCI states affect chrX gene dosage, influencing the validity of female iPSCs as model systems in studying X-linked conditions (124,125,127). For example, in Lesch-Nyhan syndrome, caused by a heterozygous mutation in the X-linked *HPRT* gene, iPSC-derived neurons showed variable traits due to reactivation of the functional *HPRT* gene from Xi. Similar anomalies have also been observed in iPSCs from patients with Rett syndrome with a mutation in the X-linked *MECP2* gene (125,127). Thus, to study sex differences, care must be taken to select stem cell lines that do not demonstrate atypical XCI states and to devise strategies to mimic XCI mosaicism in vitro.

## Limitations of Brain Organoids as an In Vitro Model of the Human Brain

Brain organoids have limitations that must be considered when using this model system. The most significant limitation is the absence of typical cortical tissue architecture. Although we observe deep-layer and upper-layer neurons, they do not have in vivo–like activity patterns, and their spatial organization is not finely tuned (35). Neuropsychiatric conditions often involve structural differences in specific cortical regions (128, 129, 130), but brain organoid models are only able to model gross dorsal and ventral forebrain traits (131). Although this works fine for studying E-I balance because excitatory and inhibitory neurons are derived from dorsal and ventral forebrain regions, respectively, during development, mechanisms that underlie human behavior such as social cognition, which is driven by subcortical regional activity, is currently beyond the scope of this method. Currently, the secondmost important limitation of brain organoids is the lack of nonneuronal cell types such as microglia. Microglia are the most significantly altered nonneuronal cell type in the autistic brain (132,133), but the development and maintenance of microglial homeostasis in the brain cannot be modeled using brain organoids because microglia are derived from the mesoderm (as opposed to the neuroectoderm for brain organoid) and do not appear to survive protracted developmental periods in vitro when cocultured (134,135). Other limitations of brain organoids include progenitor pool variability (136) and formation of necrotic tissue in larger organoids (137), but both of these limitations now have workarounds. Single-cell transcriptomic methods can help to resolve the variability in organoid progenitor pools through machine learning methods (136,138,139), while slice culture brain organoids at the air-liquid interface have been developed to overcome the influence of necrotic tissue formation (118,140). Other 3D models of brain development may also present unique advantages over brain organoids in studying sex differences (Table S2). As a result, careful consideration must be given to observable traits such as cell proliferation and E-I imbalances when using brain organoids, and if necessary, validations in other 3D model systems should be undertaken.

## Conclusions

In this review, we discussed the roles of sex steroid hormones and sex chromosomes on sex differences in brain development. Sex differences are prevalent across behavioral and neuroanatomical modalities in humans. Cellular/molecular markers of sex differences, which are more challenging to study in humans, have been revealed through animal studies. These studies have revealed multiple mechanisms of sex differences influenced by sex steroids and sex chromosomes. However, cellular phenotypes such as cell proliferation, a mechanism for brain volume differences between males and females, and E-I balance, a mechanism commonly affected in neuropsychiatric conditions with a sex bias, show species-specific variations. This has resulted in the need to develop a human model system such as stem cell–derived brain organoids. Brain organoids have already been proven effective in modeling these cellular phenotypes in X-linked neuropsychiatric conditions as well as conditions of elevated sex steroids. However, the influence of sex chromosomes on sex differences, especially chrX, is complex and remains less well understood, partially due to the regulatory activity of XCI. Recent studies have shown that XCI is only partial, and stem cell systems may be prone to atypical XCI states that affect X-linked gene dosage. The usefulness of brain organoids is also limited by their inability to recapitulate complexities of in vivo brain development; thus, other 3D in vitro models may be needed. Nonetheless, it is a flexible system that is helping us understand the biological basis of typical sex differences and sex bias in neuropsychiatric conditions.
