# Supplementary material for: Using Organoids to Model Sex Differences in the Human Brain
Source: Biol Psychiatry Glob Open Sci. 2024 Jun 4;4(5):100343. doi: 10.1016/j.bpsgos.2024.100343 (PMC11292257; doi:10.1016/j.bpsgos.2024.100343)
Supplement: Supplement [file mmc1.pdf]

SUPPLEMENTARY INFORMATION

Using Organoids to Model Sex Differences in the Human Brain

Pavlinek *et al.*

Supplementary table 1: Table of steroid effects on synapses – selected studies

Hormone/Compound	Effects of treatment on synapse parameter (treatment timeframe)	Species and age (Model)	Reference
17β-estradiol	Increased excitatory postsynaptic amplitude and LTP amplitude (acute)	Rat pups* (hippocampal slices); adult and aged male rats (hippocampal slices)	(1, 2)
	Induced NMDA-independent LTP (acute)	Young male rats (hippocampal slices)	(3)
	Enhanced induction of LTP, increased the density of parallel fiber to Purkinje cell synapses (14 days)	Ovariectomized female and male adult mice (cerebellar slices)	(4)
	Increased field excitatory postsynaptic potentials (acute), increased LTP	Young male and female rats and mice (hippocampal slices)	(5)
	Enhanced LTP, increased field excitatory postsynaptic potentials (acute)	Young male rats (hippocampal slices)	(6)
	Enhanced synaptic response to stimulation of the vestibular nerve, but reduces spontaneous discharge in A and B neurons; enhanced excitatory post-synaptic potential and current	Male rats (medial vestibular nucleus brainstem slices)	(7)
	Induced LTP, increased density of small spines (acute)	Adult male rat (hippocampal slices)	(8)
	Decreased inhibitory postsynaptic currents and suppressed GABA release in females only	Adult ovariectomized female and male rats (hippocampal slices)	(9)
	Increased field excitatory postsynaptic potentials (acute)	adult male rats (hippocampal slices);	(10, 11)
	Increased fast glutamatergic transmission, increased number of actin spines, facilitated LTP	Young male rats and ovariectomized middle-aged female rats (hippocampal slices)	(12)
	Increased excitatory postsynaptic potentials (45 minutes)	Male and female mice (hippocampal slices)	(13)
	17β-estradiol treatment rescued aromatase knockout-associated deficits: increased LTP amplitude, increased synaptophysin and PSD95 protein levels (7 days)	Female mice	(14)
	Reduced glutamate decarboxylase in interneurons, reduced miniature inhibitory postsynaptic currents (24 hrs)	Embryonic rat* (primary culture containing hippocampal interneurons)	(15)

	Increased excitatory postsynaptic currents, increased miniature excitatory postsynaptic currents, enhanced oscillation of glutamate-induced calcium	Young male and female rats (hippocampal slices)	(16)
	Increased dendritic spine density and increased synaptic surface AMPAR content, increased GluN2A-NMDAR and decreasing GluN2B-NMDAR synaptic content (24 hrs)	Rat embryos* (primary neural culture), male mice	(17)
	Increased expression of PSD-95, increased spine density	Male and female rats (hippocampal slices and primary neural cultures)	(18)
	Increased spine density, formation of silent synapses (acute)	Embryonic rat* (cortical neuronal cultures)	(19)
	Increased synaptic excitability (48hrs), increased excitatory postsynaptic potentials and potentiation of responses to AMPA, kainate, and quisqualate, but not NMDA (acute)	Ovariectomized adult female rats (hippocampal slices)	(20)
	Increased field excitatory postsynaptic potentials, increased membrane levels of GluR1 (acute)	Embryonic rats* and mice* (cultured cortical and hippocampal neurons, acute hippocampal slices)	(21)
	Enhanced release of dopamine	Ovariectomized female rats (dorsolateral striatum)	(22)
Aromatase inhibitor (Letrozole)	Prevented LTP at fiber–Purkinje cell synapses (acute)	Adult male rats (cerebellar slices)	(23)
	Reduced basolateral amygdala spine synapse density in female mice only (7 days), prevented LTP induction in females only (60 minutes)	Male and female mice, male and female neonatal rats (corticoamygdalar slices)	(24)
Dihydrotestosterone	Increased spine synapse density (2 days)	ovariectomized adult female rats (hippocampus)	(25)
Estradiol benzoate	Increased field excitatory postsynaptic potentials (acute)	Adult ovariectomized female rats (hippocampal slices)	(26)
Flutamide (AR blocker)	Prevented LTD, No effect on LTP (acute)	Male rats (brainstem slices); Young and adult male rats (hippocampal slices)	(27, 28)
ICI 182,780 (ER blocker)	Prevented LTP, No effect on LTD	Male rats (brainstem slices); Young and adult	(27, 28)

		male rats (hippocampal slices)	
MPP (ERα Antagonist) and ERβ Antagonist (PHTPP)	Reduced LTP amplitude, in combination prevented LTP completely (acute)	Young and adult male rats (hippocampal slices)	(28)
PPT (ERα agonist)	Induced LTP, increased GluA1 surface staining (acute)	Cultured hippocampal neonatal male rat neurons	(3)

LTP - long-term potentiation

LTD - long-term depression

* sex not reported/not considered

acute treatment - <1 hour

effects on synapses – effects on electrophysiological parameters, synaptic proteins, synaptic puncta and spines

Supplementary table 2: Table of brain-region specific organoids

Organoid type	Advantages	Reference
Cortical spheroid	Dorsal or ventral forebrain spheroids that can be assembled *in vitro* to recapitulate fetal migration of interneurons and their functional integration with glutamatergic neurons. This model of cortical development follows a targeted approach that specifically studies interaction of excitatory and inhibitory neurons, and can provide a better understanding of E-I imbalance mechanisms.	(29)
Hippocampal organoid	Recapitulates hippocampus development, a key brain region influenced by sex steroids. Generates hippocampal granule-like, pyramidal-like neurons, and astrocyte-like cells.	(30)
Cerebellar organoid	Recapitulates early cerebellar development, the cerebellum being a non-cortical brain region showing sex-specific development. Generates precursors of cerebellar neurons (Purkinje-like, Golgi-like, DCN projection-like neurons, granule-like cells) with relevant morphology and electrophysiological properties.	(31)

References

1. Bi R, Broutman G, Foy MR, Thompson RF, Baudry M (2000): The tyrosine kinase and mitogen-activated protein kinase pathways mediate multiple effects of estrogen in hippocampus. *Proc Natl Acad Sci U S A*. 97:3602-3607.
2. Foy MR, Baudry M, Foy JG, Thompson RF (2008): 17beta-estradiol modifies stress-induced and age-related changes in hippocampal synaptic plasticity. *Behav Neurosci*. 122:301-309.
3. Clements L, Harvey J (2020): Activation of oestrogen receptor α induces a novel form of LTP at hippocampal temporoammonic-CA1 synapses. *British Journal of Pharmacology*. 177:642-655.
4. Corina EA, Bogdan AM, Elize DH, Piet K, Frank HDJ, Andrée K, et al. (2007): Estradiol Improves Cerebellar Memory Formation by Activating Estrogen Receptor β. *The Journal of Neuroscience*. 27:10832.
5. Di Mauro M, Tozzi A, Calabresi P, Pettorossi V, Grassi S (2015): Neo-synthesis of estrogenic or androgenic neurosteroids determine whether long-term potentiation or depression is induced in hippocampus of male rat. *Frontiers in Cellular Neuroscience*. 9.
6. Foy MR, Xu J, Xie X, Brinton RD, Thompson RF, Berger TW (1999): 17β-Estradiol Enhances NMDA Receptor-Mediated EPSPs and Long-Term Potentiation. *Journal of Neurophysiology*. 81:925-929.
7. Grassi S, Frondaroli A, Scarduzio M, Dutia MB, Dieni C, Pettorossi VE (2010): Effects of 17B-estradiol on glutamate synaptic transmission and neuronal excitability in the rat medial vestibular nuclei. *Neuroscience*. 165:1100-1114.
8. Hasegawa Y, Hojo Y, Kojima H, Ikeda M, Hotta K, Sato R, et al. (2015): Estradiol rapidly modulates synaptic plasticity of hippocampal neurons: Involvement of kinase networks. *Brain Research*. 1621:147-161.
9. Huang Guang Z, Woolley Catherine S (2012): Estradiol Acutely Suppresses Inhibition in the Hippocampus through a Sex-Specific Endocannabinoid and mGluR-Dependent Mechanism. *Neuron*. 74:801-808.
10. Wang W, Kantorovich S, Babayan AH, Hou B, Gall CM, Lynch G (2016): Estrogen's Effects on Excitatory Synaptic Transmission Entail Integrin and TrkB Transactivation and Depend Upon β1-integrin function. *Neuropsychopharmacology*. 41:2723-2732.
11. Kim MT, Soussou W, Gholmieh G, Ahuja A, Tanguay A, Berger TW, Brinton RD (2006): 17β-Estradiol potentiates field excitatory postsynaptic potentials within each subfield of the hippocampus with greatest potentiation of the associational/commissural afferents of CA3. *Neuroscience*. 141:391-406.
12. Kramár EA, Chen LY, Brandon NJ, Rex CS, Liu F, Gall CM, Lynch G (2009): Cytoskeletal changes underlie estrogen's acute effects on synaptic transmission and plasticity. *Journal of Neuroscience*. 29:12982-12993.
13. Kumar A, Bean LA, Rani A, Jackson T, Foster TC (2015): Contribution of estrogen receptor subtypes, ERα, ERβ, and GPER1 in rapid estradiol-mediated enhancement of hippocampal synaptic transmission in mice. *Hippocampus*. 25:1556-1566.
14. Lu Y, Sareddy GR, Wang J, Wang R, Li Y, Dong Y, et al. (2019): Neuron-Derived Estrogen Regulates Synaptic Plasticity and Memory. *The Journal of Neuroscience*. 39:2792-2809.

15. Murphy DD, Cole NB, Greenberger V, Segal M (1998): Estradiol increases dendritic spine density by reducing GABA neurotransmission in hippocampal neurons. *J Neurosci*. 18:2550-2559.
16. Oberlander JG, Woolley CS (2017): 17β-Estradiol Acutely Potentiates Glutamatergic Synaptic Transmission in the Hippocampus through Distinct Mechanisms in Males and Females. *The Journal of Neuroscience*. 37:12314-12327.
17. Potier M, Georges F, Brayda-Bruno L, Ladépêche L, Lamothe V, Al Abed AS, et al. (2016): Temporal Memory and Its Enhancement by Estradiol Requires Surface Dynamics of Hippocampal CA1 N-Methyl-D-Aspartate Receptors. *Biol Psychiatry*. 79:735-745.
18. Sato K, Akaishi T, Matsuki N, Ohno Y, Nakazawa K (2007): β-Estradiol induces synaptogenesis in the hippocampus by enhancing brain-derived neurotrophic factor release from dentate gyrus granule cells. *Brain Research*. 1150:108-120.
19. Srivastava DP, Woolfrey KM, Jones KA, Shum CY, Lash LL, Swanson GT, Penzes P (2008): Rapid enhancement of two-step wiring plasticity by estrogen and NMDA receptor activity. *Proc Natl Acad Sci U S A*. 105:14650-14655.
20. Wong M, Moss RL (1992): Long-term and short-term electrophysiological effects of estrogen on the synaptic properties of hippocampal CA1 neurons. *J Neurosci*. 12:3217-3225.
21. Zadran S, Qin Q, Bi X, Zadran H, Kim Y, Foy MR, et al. (2009): 17-β-Estradiol increases neuronal excitability through MAP kinase-induced calpain activation. *Proceedings of the National Academy of Sciences*. 106:21936-21941.
22. Zhimin S, Hongyan Y, Elizabeth MP, Jill BB (2019): Estradiol-Induced Potentiation of Dopamine Release in Dorsal Striatum Following Amphetamine Administration Requires Estradiol Receptors and mGlu5. *eneuro*. 6:ENEURO.0446-0418.2019.
23. Dieni CV, Ferraresi A, Sullivan JA, Grassi S, Pettorossi VE, Panichi R (2018): Acute inhibition of estradiol synthesis impacts vestibulo-ocular reflex adaptation and cerebellar long-term potentiation in male rats. *Brain Structure and Function*. 223:837-850.
24. Roland AB, Lepu Z, Ricardo V, Nicola B, Alexander K, Christine EG, et al. (2017): Sex-Dependent Regulation of Aromatase-Mediated Synaptic Plasticity in the Basolateral Amygdala. *The Journal of Neuroscience*. 37:1532.
25. Leranth C, Hajszan T, J. MacLusky N (2004): Androgens Increase Spine Synapse Density in the CA1 Hippocampal Subfield of Ovariectomized Female Rats. *The Journal of Neuroscience*. 24:495.
26. Sharrow KM, Kumar A, Foster TC (2002): Calcineurin as a potential contributor in estradiol regulation of hippocampal synaptic function. *Neuroscience*. 113:89-97.
27. Scarduzio M, Panichi R, Pettorossi VE, Grassi S (2013): Synaptic Long-Term Potentiation and Depression in the Rat Medial Vestibular Nuclei Depend on Neural Activation of Estrogenic and Androgenic Signals. *PLOS ONE*. 8:e80792.
28. Tozzi A, Durante V, Manca P, Di Mauro M, Blasi J, Grassi S, et al. (2019): Bidirectional Synaptic Plasticity Is Driven by Sex Neurosteroids Targeting Estrogen and Androgen Receptors in Hippocampal CA1 Pyramidal Neurons. *Frontiers in Cellular Neuroscience*. 13.
29. Birey F, Andersen J, Makinson CD, Islam S, Wei W, Huber N, et al. (2017): Assembly of functionally integrated human forebrain spheroids. *Nature*. 545:54-59.

30. Sakaguchi H, Kadoshima T, Soen M, Narii N, Ishida Y, Ohgushi M, et al. (2015): Generation of functional hippocampal neurons from self-organizing human embryonic stem cell-derived dorsomedial telencephalic tissue. *Nature Communications*. 6:8896.
31. Muguruma K, Nishiyama A, Kawakami H, Hashimoto K, Sasai Y (2015): Self-Organization of Polarized Cerebellar Tissue in 3D Culture of Human Pluripotent Stem Cells. *Cell Reports*. 10:537-550.
